# The efficacy and safety of serratus anterior plane block in patients undergoing cardiac surgery: A systematic review and meta-analysis

**DOI:** 10.1097/MD.0000000000048013

**Published:** 2026-03-13

**Authors:** Xu-Bo Cui, Ming-Zhu Cui, Qiu-Ju Yang, Yang Liu, Meng-Qi Yi, Xiao-Yang Zhang, Yun-Tai Yao

**Affiliations:** a Department of Anesthesiology and Perioperative Medicine, People’s Hospital of Henan University, Henan Provincial People’s Hospital, Zhengzhou, China; b Department of Anesthesiology and Perioperative Medicine, People’s Hospital of Zhengzhou University, Henan Provincial People’s Hospital, Zhengzhou, Henan, China; c Department of Anesthesiology, Fuwai Hospital, National Center for Cardiovascular Diseases, Peking Union Medical College and Chinese Academy of Medical Sciences, Beijing, China; d Evidence in Cardiovascular Anesthesia (EICA) Group, Beijing, China; e Department of Anesthesiology, Center of Outcomes Research, Critical Care and Pain Medicine, University of Texas, Houston, TX; f Outcomes research Consortium, Houston, TX.

**Keywords:** cardiac surgery, efficacy, opioid analgesics, pain management, safety, serratus anterior plane block

## Abstract

**Background:**

Serratus anterior plane block (SAPB) is a widely used fascial block that reduces postoperative pain and perioperative opioid consumption. This meta-analysis assessed the efficacy and safety of SAPB in cardiac surgery patients.

**Methods:**

We systematically searched PubMed, Web of Science, Cochrane Library, Embase, China National Knowledge Infrastructure, and Wanfang databases on May 10, 2024. Pooled relative risks and mean differences (MD) with 95% confidence intervals (CI) were calculated for dichotomous and continuous outcomes, respectively. Heterogeneity was assessed using the *I*^2^ statistic, and publication bias was evaluated using Egger test. Subgroup analyses were stratified by study design (randomized controlled trials vs cohort studies) and patient age to explore methodological heterogeneity.

**Results:**

Fifteen studies, encompassing 1169 adult and pediatric participants, were included. Overall, compared to general anesthesia (GA) alone, the SAPB + GA group significantly reduced intensive care unit length of stay (MD = −6.61 hours, 95% CI: −10.91 to −2.32), decreased postoperative analgesic consumption (MD = −4.20 mg morphine equivalents, 95% CI: −6.52 to −1.88), and lowered the risk of complications (relative risk = 0.63, 95% CI: 0.40–0.99). SAPB + GA also lowered postoperative Visual Analogue Scale pain scores (MD = −1.25, 95% CI: −1.74 to −0.75) and serum cortisol levels (MD = −35.43 nmol/L, 95% CI: −58.58 to −12.27). No local anesthetic toxicity or mortality was reported.

**Conclusions:**

Perioperative SAPB combined with GA provides significant benefits in cardiac surgery, including accelerated intensive care unit discharge, reduced opioid use and pain scores, attenuated stress response (reduced cortisol), and favorable safety, which supports enhanced patient recovery.

## 1. Introduction

Patients undergoing cardiac surgery frequently experience significant postoperative pain, which can impede recovery and prolong hospitalization.^[1]^ Perioperative pain management in these procedures often requires substantial opioid administration,^[2]^ typically delivered via oral, intravenous, or patient-controlled analgesia routes.^[3,4]^ However, high-dose opioid use may prolong hospital stays or delay extubation, exposing patients to additional risks. Consequently, effective acute postoperative pain management and opioid reduction are critical priorities.^[5,6]^ Importantly, the risk of acute pain progressing to chronic pain remains clinically significant following cardiac surgery.

The concept of enhanced recovery after surgery involves the standardization of patient care protocols through multimodal, interdisciplinary treatment plans, aiming to improve surgical outcomes and achieve higher-quality patient recovery.^[7]^ Regional anesthesia techniques, including thoracic epidural analgesia and paravertebral block, have been integrated with general anesthesia (GA) to mitigate postoperative pain following minimally invasive chest surgery. Given that heparinization is frequently necessary during perioperative cardiac procedures, the utilization of paravertebral block and thoracic epidural analgesia inherently entails risk of epidural hematoma or hemorrhage.^[8]^ Among the diverse nerve block methodologies, fascial-plane blocks are emerging as a viable alternative to conventional techniques such as paravertebral and epidural blocks.^[9]^ Furthermore, there has been an increasing body of research and case reports examining techniques for interfacial nerve blocks, which have generated heightened interest in their application during cardiac surgery procedures.^[10]^ The ultrasound-guided serratus anterior plane block (SAPB) is emerging as a prominent fascial-plane block due to its simplicity, safety, and efficacy in providing analgesia in the same side of the chest.^[11,12]^

The SAPB is a widely utilized local muscle-fascial block technique that has demonstrated effectiveness in alleviating patients’ postoperative acute pain and reducing perioperative opioid consumption.^[13]^ This technique involves the administration of local anesthetics into the interstitial space between the serratus anterior and latissimus dorsi muscles to alleviate chest discomfort. It has been extensively utilized in breast surgery, thoracotomy, and rib surgery, demonstrating significant efficacy in reducing postoperative pain.^[14–16]^ However, there is a paucity of evidence concerning the postoperative analgesic effects of SAPB in patients undergoing cardiac surgery. Therefore, we conducted a systematic review and meta-analysis of randomized controlled trials (RCTs) and cohort studies to evaluate the analgesic efficacy of SAPB following cardiac surgery, specifically considering its efficacy in relation to side effects.

## 2. Methods

### 2.1. Ethical approval

This study was a meta-analysis of previously published studies. Therefore, ethical approval was not required according to the policies of the Ethical Committee of Henan Provincial People’s Hospital.

### 2.2. Search strategy

This comprehensive review was conducted in accordance with the guidelines of the Preferred Reporting Items for Systematic Reviews and Meta-Analysis (PRISMA).^[17]^ The protocol for the present meta-analysis has been registered with the International Prospective Systematic Reviews Registry (PROSPERO: CRD42024507291). We systematically searched PubMed, Cochrane Library, Embase, Web of Science, CNKI, and Wanfang databases from inception to May 10, 2024. The search terms employed included: “Cardiac Surgical Procedures,” “Coronary Artery Bypass,” “Extracorporeal Circulation,” “Cardiac Diseases,” “Cardiopulmonary Bypass,” and “SAPB.” We also meticulously screened the references of the identified articles. The search was not restricted by language. Two independent authors (XBC and MZC) conducted the selection of trials based on predefined eligibility criteria. The search strategies employed for each database are comprehensively detailed in the supplementary materials (Table S1, Supplemental Digital Content, https://links.lww.com/MD/R515).

### 2.3. Inclusion and exclusion criteria

The inclusion criteria encompassed all studies investigating the use of SAPB in cardiac surgery, specifically including RCTs, retrospective cohort studies, and prospective cohort studies. Studies were excluded if: SAPB was not administered in conjunction with GA; outcome data were inaccessible and attempts to contact the authors were unsuccessful; and the studies were unpublished, ongoing, or available only as conference abstracts.

This meta-analysis involved a comparative analysis between the SAPB group and a GA group, which included patients who did not receive a block, patient-controlled analgesia, or continuous wound infiltration. The population (P) comprised patients undergoing cardiac surgical procedures. The intervention (I) was the SAPB, utilized as an adjunctive pain relief method, administered either pre- or post-induction of GA (SAPB + GA group). The control (C) was the GA group (GA group). The study outcomes (O) encompassed at least one of the primary or secondary endpoints specified below. The study design (S) included RCTs and cohort studies.

### 2.4. Data abstraction

Two independent researchers (YL and QJY) systematically collected data from each trial, including the 1st author, year of publication, sample size, patient characteristics, specifics of the SAPB, postoperative analgesia, types of surgical procedures, and reported outcomes. Opioid consumption was standardized to intravenous morphine equivalents using a conversion table (Table S1, Supplemental Digital Content, https://links.lww.com/MD/R516).

The length of stay (LOS), which serves as a tangible manifestation of both the overall efficacy of treatment and the efficiency of hospital resource utilization, was designated as the primary outcome. Specifically, the LOS in the hospital or intensive care unit (ICU) was the key parameter under assessment, with all other outcomes being classified as secondary ones.

The secondary outcomes assessed encompassed the incidence of postoperative complications, postoperative opioid consumption, and postoperative pain scores. Pain assessment was conducted using the Face, Legs, Activity, Crying, Consolability (FLACC) scores, static and dynamic pain visual analogue scale (VAS) scores, and numerical rating scale (NRS) scores. Additionally, the postoperative stress state was evaluated through serum levels of cortisol and interleukin. Patient satisfaction with analgesia was measured on the 1st postoperative day using an 11-point scale, where 0 indicated complete dissatisfaction and 10 indicated complete satisfaction.

### 2.5. Study quality assessment

The methodological quality of the included RCTs was appraised utilizing the Risk of Bias Summary Tool from the Cochrane Collaboration.^[18]^ For the non-randomized cohort studies, quality assessment was conducted using the Newcastle–Ottawa Scale, which evaluates the selection of study groups, the comparability between groups, and the ascertainment of outcomes. The processes of literature search, data extraction, risk of bias assessment, and quality evaluation were independently performed by 3 researchers (XBC, MZC, and MQY). Any disagreements or discrepancies were resolved through consensus with a third author (XYZ).

### 2.6. Statistical analysis

Analyses used RevMan 5.4 (The Cochrane Collaboration, London) and STATA 18.0 (StataCorp LLC, College Station). Dichotomous data are reported as relative risks (RR) with 95% confidence intervals (CI), continuous data as mean differences (MD) with 95% CI. Heterogeneity was quantified using *I*^2^ (*I*^2^ = 25–49%: low; 50–74%: moderate; ≥75%: high). Due to the inherent clinical and methodological heterogeneity across studies in surgery and anesthesiology, the use of a random-effects model for the meta-analysis was warranted. Statistical significance was set at *P* < .05. Potential publication bias was examined by Egger test, and the robustness of the pooled results was evaluated through leave-one-out sensitivity analysis, respectively.

## 3. Results

### 3.1. Search results

**Figure 1.**
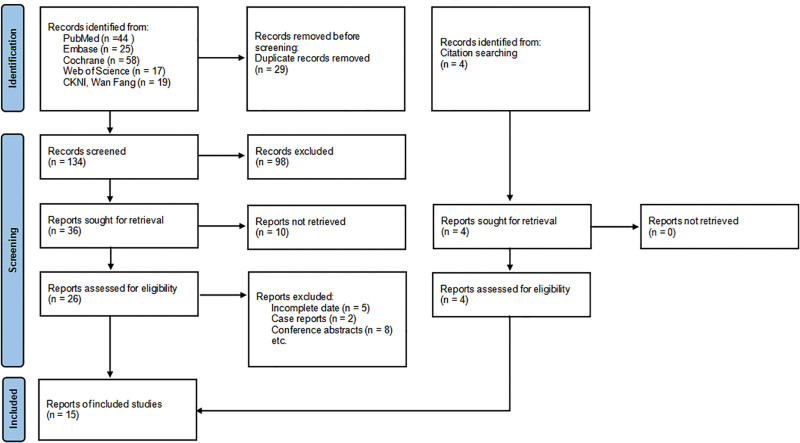
PRISMA flow diagram. PRISMA = Preferred Reporting Items for Systematic Reviews and Meta-Analysis. Source: Page et al^[19]^

**Figure 2.**
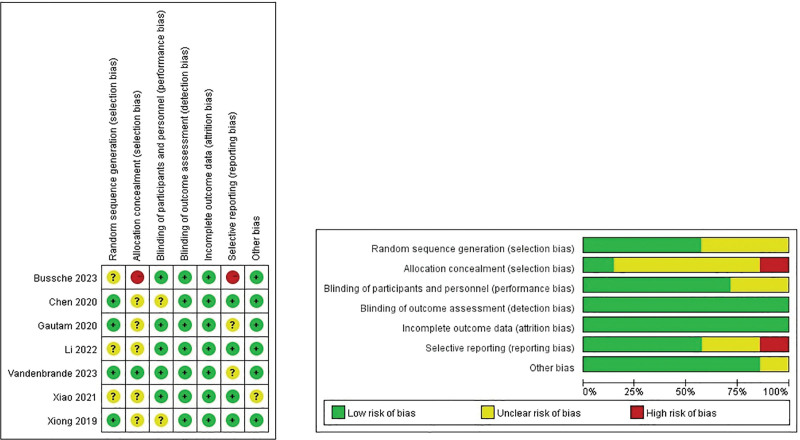
Risk of bias summary and graph.

The screening process is illustrated in the Preferred Reporting Items for Systematic Reviews and Meta-Analysis diagram (Fig. 1), which delineates the number of articles included and excluded at each stage of the bibliographic search. The characteristics of the incorporated studies are detailed in Table 1. The results of the risk of bias assessment are presented in Figure 2 and Table 2. Initially, 167 potentially relevant articles were identified. After the removal of duplicates, 138 articles remained. Subsequently, 123 articles were excluded following the screening of titles, abstracts, and full texts. Finally, 7 RCTs and 8 cohort studies met the inclusion criteria.^[20–34]^ These studies,^[20–26]^ comprising both retrospective and prospective cohort trials,^[27–34]^ involved a total of 1169 participants. The studies enrolled both adult and pediatric patients, and baseline data such as weight and age were analyzed. No significant statistical variances were observed between the 2 groups (Figure S1, Supplemental Digital Content, https://links.lww.com/MD/R516).

**Table 1:** Study characteristics of the included trials.

Study	Sample size		Patients’ characteristics						SAPB techniques			Postoperative analgesia	Surgery	Reported outcomes
			Age (yr)		Sex (F/M)		Weight (kg)/BMI		Timing	Guided	Local anesthetic			
	GS	GG	GS	GG	GS	GG	GS	GG						
Gautam et al^[20]^	25	25	62 ± 9	59 ± 11	2/23	3/22	66.5 ± 12.8	58.9 ± 11.1	Postoperative	US	20 mL mixture of 0.2% ropivacaine with 1 µg/mL fentanyl	No block	Minimally invasive CABG	①②③⑤⑦
Vandenbrande et al^[21]^	38	37	72 ± 9	67 ± 10	13/25	15/22	27.4 ± 3.3 (BMI)	28.1 ± 3.8 (BMI)	Postoperative	US	0.25% ropivacaine 40 mL	PCA	Endoscopic AVR	①②③⑥⑦⑨
Bussche et al^[22]^	21	26	58 ± 21	64 ± 18	10/11	17/9	24.7 ± 3.2 (BMI)	26.8 ± 4.1 (BMI)	Postoperative	US	0.2% ropivacaine 10 mL/h continuous infusion	CWI	Minimally invasive MVR	①②③⑦
Xiong and Chen^[23]^	20	20	36 ± 9	38 ± 10	7/13	8/12	23.8 ± 3.3 (BMI)	23.4 ± 3.5 (BMI)	Preoperative	US	0.5% ropivacaine 20 mL	PCA	Thoracoscopic heart surgery	①②③⑤⑦⑧
Li et al^[24]^	40	40	3 ± 2	3 ± 2	20/20	23/17	13.8 ± 3.7	14.0 ± 4.3	Preoperative	US	0.375% ropivacaine 3 mL/kg	No block	VSD	①③
Chen and Ren^[25]^	50	50	4 ± 3	4 ± 2	26/24	20/30	12.1 ± 4.1	12.8 ± 4.7	Preoperative	US	0.2% ropivacaine 3 mL/kg	No block	ASD VSD PDA	②③④
Xiao and Ma^[26]^	34	34	3 ± 1	3 ± 1	16/18	18/16	14.3 ± 3.4	14.0 ± 3.2	Preoperative	US	0.3% bupivacaine 0.5 mL/kg	PCA	ASD VSD	②④
Aykut et al^[27]^	43	56	61 ± 10	61 ± 8	8/35	11/45	28.6 ± 3.8 (BMI)	28.8 ± 4.2 (BMI)	Preoperative	US	0.5% bupivacaine 40 mL	Standard treatment of pain	CABG	①②③⑤⑦
Berthoud et al^[28]^	20	26	67 ± 11	67 ± 6	10/10	8/18	27.6 ± 6.4 (BMI)	27.0 ± 3.1 (BMI)	Postoperative	US	0.5% ropivacaine 200 mg	CWI	Minimally invasive cardiac surgery	①②③⑥⑦
Saikat et al^[29]^	40	40	63 ± 9	60 ± 11	N/A	N/A	24.1 ± 1.8 (BMI)	24.8 ± 2.1 (BMI)	Postoperative	US	0.2% ropivacaine 20 mL with continued infusion 20 mg/h	Intravenous fentanyl infusion	Minimally invasive cardiac surgery	①②⑤⑧
Toscano et al^[30]^	33	26	60 ± 16	64 ± 14	17/16	14/12	68.9 ± 13.3	65.5 ± 11.0	Preoperative	US	0.375% ropivacaine 150 mg with continuous infusion 21 mg/h	PCA	MVR Performed in right mini thoracotomy	①②③⑥⑦
Yasar et al^[31]^	32	33	61 ± 11	61 ± 9	3/29	7/26	83.4 ± 10.3	80.1 ± 10.8	Postoperative	US	0.25% bupivacaine 0.5 mL/kg	PCA	Isolated Bypass Surgery	②③⑦
Wang and Wang^[32]^	34	46	68 ± 2	68 ± 2	16/18	22/24	21.1 ± 1.0 (BMI)	21.1 ± 1.0 (BMI)	Preoperative	US	0.5% ropivacaine 20 mL	PCA	VSD with pulmonary hypertension	②⑧
Toscano et al^[33]^	32	22	60 ± 16	62 ± 14	17/15	11/11	24.7 ± 3.9 (BMI)	23.2 ± 3.6 (BMI)	Postoperative	N/I	0.375% ropivacaine 150 mg with continuous infusion of ropivacaine 0.3% 21 mg/h	Intravenous morphine infusion	MVR Performed in right mini thoracotomy	①
Moll et al^[34]^	116	110	63 ± 12	66 ± 12	27/89	23/87	29.6 ± 5.2 (BMI)	28.4 ± 4.2 (BMI)	Postoperative	US	0.375% bupivacaine 25 mL with continuous infusion 0.2% bupivacaine 10 mg/h	No block	CABG	①

① Length of stay in the hospital or intensive care unit; ② the incidence of postoperative complications; ③ postoperative opioid consumption; ④ Face, Legs, Activity, Crying, Consolability (FLACC) scores; ⑤ Visual analogue scale (VAS) scores; ⑥ numerical rating scale (NRS) scores; ⑦ postoperative opioid consumption; ⑧ postoperative stress state; ⑨ patients’ satisfaction with analgesia.

ASD = atrial septal defect, AVR = aortic valve replacement, BMI = body mass index, CABG = coronary artery bypass surgery, CWI = continuous wound infiltration, GA = general anesthesia, GG = group GA, GS = group SAPB + GA, MVR = mitral valve replacement, N/A = not applicable, N/I = no information, PCA = patient-controlled analgesia, PDA = patent ductus arteriosus, SAPB = serratus anterior plane block group, US = ultrasound, VSD = ventricular septal defect.

**Table 2:** Quality assessment of the included studies.

Study	Representativeness of the exposed cohort	Selection of the nonexposed cohort	Ascertainment of exposure	Demonstration that outcome of interest of was not present at start of study	Comparability of cohorts on the basis of the design or analysis	Assessment of outcome	Was follow-up long enough for outcomes to occur	Adequacy of follow-up the cohorts	Quality scores
Toscano et al^[30]^	★	★	★	★	★★	★	★	★	9
Toscano et al^[33]^	★	★	★	★	★★	★	★	★	9
Yasar et a^[31]^	★	★	★	★	★	★		★	7
Saikat et al^[29]^	★	★	★	★	★	★	★	★	8
Moll et al^[34]^	★	★	★	★	★	★	★	★	8
Aykut et al^[27]^	★	★	★	★	★★	★	★	★	9
Wang and Wang^[32]^	★	★	★	★	★★	★	★	★	9
Berthoud et al^[28]^	★	★	★	★	★★	★	★	★	9

### 3.2. LOS

The LOS in the hospital was recorded in 9 studies, which included 4 RCTs and 5 cohort studies.^[20–22,24,27,28,30,33,34]^ One of the 9 studies focused on pediatric patients, while the remaining 8 studies involved adult participants. We assessed the LOS following surgery. The SAPB + GA group and the GA group each comprised 327 patients. A pooled analysis indicated no statistically significant difference in LOS between the 2 groups (MD = −0.01, 95% CI: −0.43 to 0.40, *P* = *.96*, *I*^2^ = 45%). Li et al^[24]^ was the only study to report LOS in the ICU or hospital for pediatric patients post-surgery, with no significant difference observed (*P* *>* .05).

**Figure 3.**
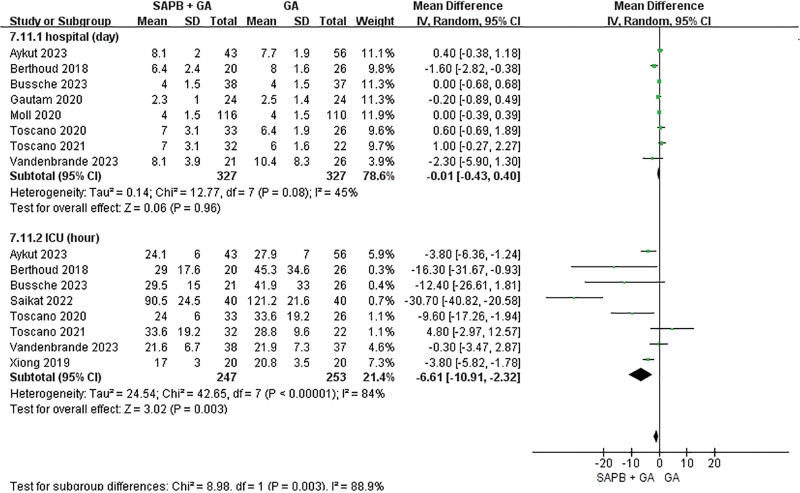
Forest plot of the length of stay in the hospital or intensive care unit.

Eight studies provided data on postoperative LOS in the ICU.^[21–23,27–30,33]^ The LOS in the ICU was significantly reduced in the SAPB + GA group compared to the GA group (MD = −6.61, 95% CI: −10.91 to −2.32, *P* = .003, *I*^2^ = 84%) (Fig. 3). Due to the significant heterogeneity among the studies, a subgroup analysis was performed based on study type. Three RCTs did not show a statistically significant difference between the 2 groups (MD = −2.83, 95% CI: −6.41 to 0.76, *P* = .12, *I*^2^ = 60%). Conversely, 5 cohort studies revealed statistically significant differences between the groups (MD = −10.25, 95% CI: −19.93 to 0.57, *P* = .04, *I*^2^ = 89%) (Figure S2, Supplemental Digital Content, https://links.lww.com/MD/R516).

### 3.3. The incidence of postoperative complications

**Figure 4.**
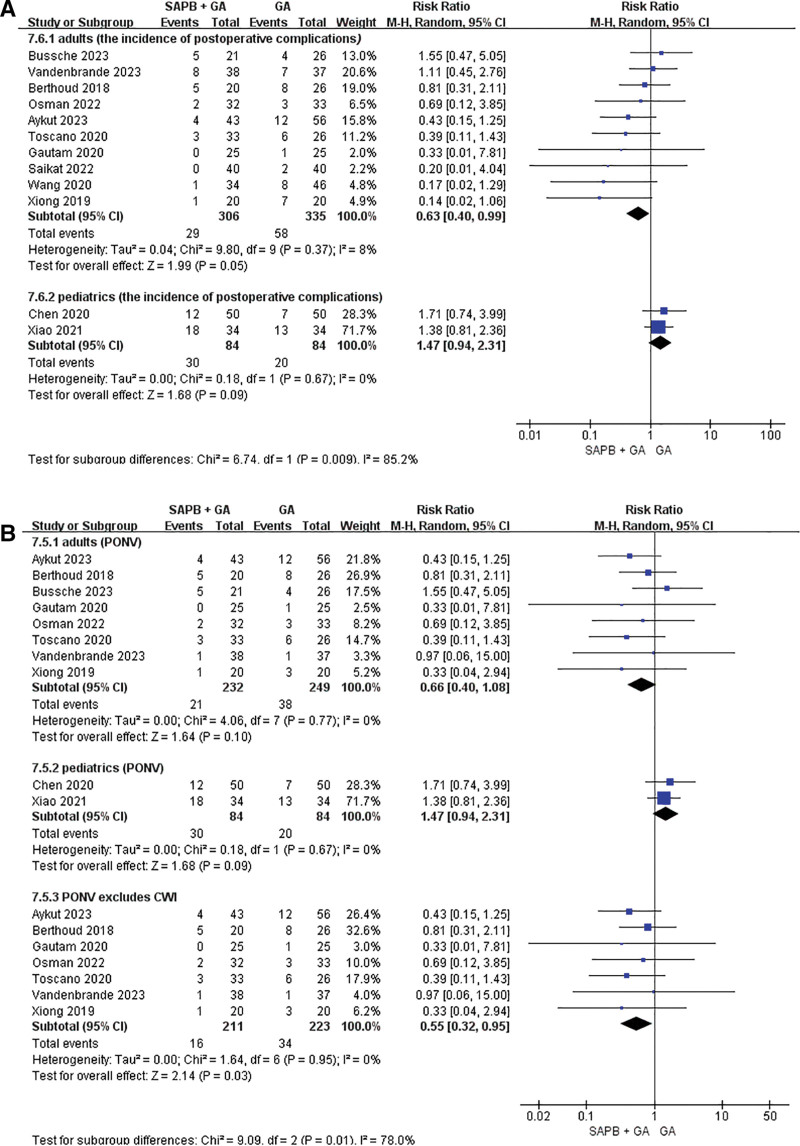
Forest plot of the incidence of postoperative complications: the incidence of total postoperative complications in adults and pediatrics (A). The incidence of postoperative nausea and vomiting in adults and pediatrics (B).

Ten studies, enrolling a total of 641 adult patients,^[20–23,27–32]^ reported the incidence of postoperative complications following cardiac surgery. These complications included postoperative nausea and vomiting (PONV), pruritus, hypoxemia, and pulmonary infection. A comparison between the SAPB + GA group and the GA group revealed that the former experienced significantly fewer complications than the latter (RR = 0.63, 95% CI: 0.40–0.99, *P* = .05, *I*^2^ = 8%). Due to the limited number of articles addressing postoperative pruritus, hypoxemia, and pulmonary infections in patients, we only conducted a subgroup analysis of PONV. The incidence of PONV was specifically reported in 8 studies,^[20–23,27,28,30,31]^ encompassing 471 patients, and no significant differences were observed between the 2 groups in the adult cohort (RR = 0.63, 95% CI: 0.39 to 1.03, *P* = .06, *I*^2^ = 0%). The probability of PONV in the SAPB + GA group was lower than that in the GA group when the references comparing continuous wound infiltration was excluded. Postoperative complications did not exhibit any statistically significant differences among pediatric patients (adults’ group: RR = 0.55, 95% CI: 0.32–0.95, *P* = .03, *I*^2^ = 0%; pediatric group: RR = 1.47, 95% CI: 0.94–2.31, *P* = .09, *I*^2^ = 0%). None of the studies reported cases of local anesthetic toxicity or fatalities (Fig. 4).

### 3.4. Postoperative pain scores

A total of 9 studies recorded postoperative pain scores of patients, including VAS scores, FLACC scores, and NRS scores. Two studies involving pediatric patients utilized FLACC scores,^[25,26]^ while 4 studies involving adult patients employed VAS pain scores,^[20,23,27,29]^ and 3 studies used NRS scores.^[21,28,30]^ FLACC scores were assessed at various intervals within the 1st 24 hours post-surgery in pediatric patients. A forest plot analysis demonstrated that SAPB significantly reduced postoperative pain, particularly at 12 and 24 hours after surgery (2 hours: MD = −1.04, 95% CI: −2.12 to 0.04, *P* = .06, *I*^2^ = 96%; 12 hours: MD = −0.51, 95% CI: −1.0 to −0.03, *P* = .04, *I*^2^ = 65%; 24 hours: MD = −0.26, 95% CI: −0.5 to −0.03, *P* = .03, *I*^2^ = 0%).

The VAS scores were utilized in 4 studies to measure both static and dynamic pain scores. These studies encompassed 128 patients in the SAPB + GA group and 141 patients in the GA group, with pain assessments conducted 24 hours post-surgery. Significant differences were observed between the SAPB + GA group and the GA group in both static and dynamic pain scores (static pain scores: MD = −1.25, 95% CI: −1.74 to −0.75, *P* *<* .00001, *I*^2^ = 63%; dynamic pain scores: MD = −1.14, 95% CI: −1.46 to −0.81, *P* *<* .00001, *I*^2^ = 0%).

**Figure 5.**
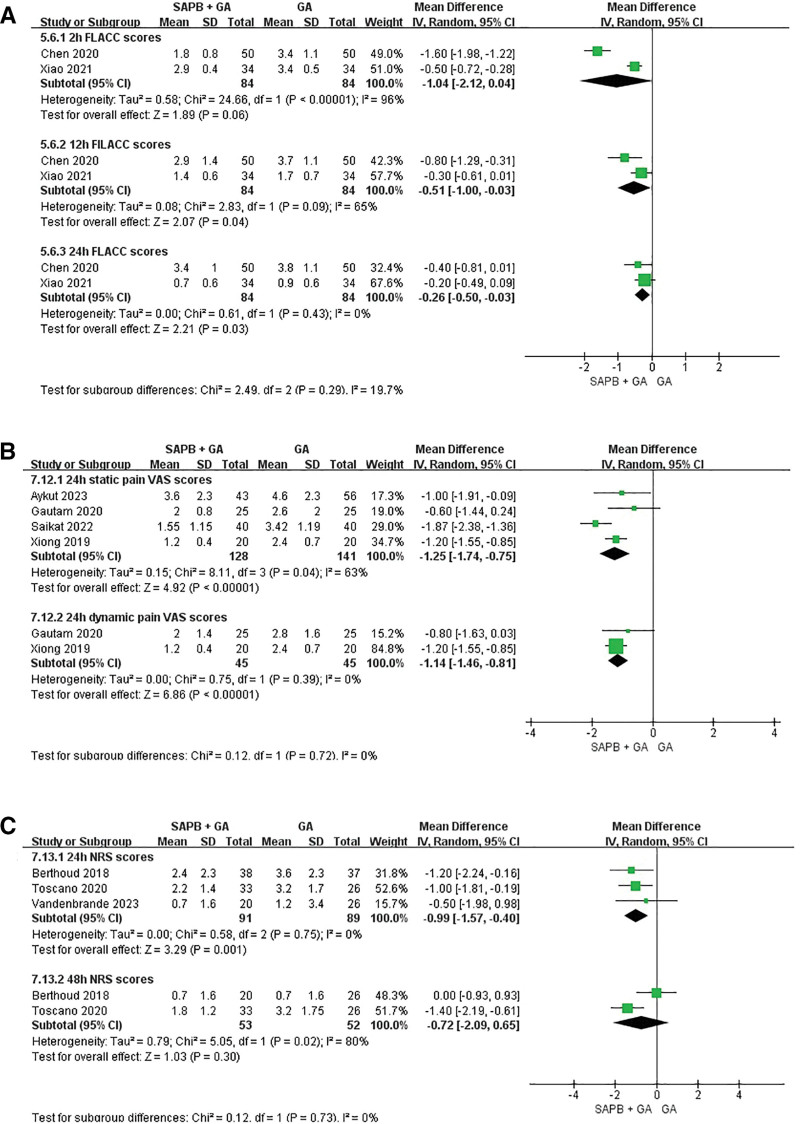
Forest plot of postoperative pain scores: the FLACC scores were assessed during the 1st postoperative 24 h in pediatrics (A). Static and dynamic pain visual analogue scale scores were assessed at 24 h postoperatively in adults (B). Numerical rating scale scores were assessed at either 24 h or 48 h postoperatively in adults (C). FLACC = Face, Legs, Activity, Crying, Consolability.

NRS scores were employed in 3 adult cohort studies, encompassing 91 patients in the SAPB + GA group and 89 patients in the GA group for the analysis of 24 hours NRS scores. The findings indicated that patients in the SAPB + GA group experienced significantly lower pain scores compared to those in the GA group (MD = −0.99, 95% CI: −1.57 to −0.40, *P* = .001, *I*^2^ = 0%). Conversely, no significant difference was observed in the 48 hours NRS scores (MD = −0.72, 95% CI: −2.09 to 0.65, *P* = .30, *I*^2^ = 80%) (Fig. 5).

### 3.5. Postoperative opioid consumption

Postoperative opioid consumption was reported in 8 studies involving adult patients.^[20–23,27,28,30,31]^ In these studies, a combination of 3 analgesics-fentanyl, tramadol, and morphine, was administered. Morphine doses were used to quantify total opioid consumption.^[35,36]^ Within 24 hours after the SAPB was administered, the forest plot revealed that the SAPB + GA group exhibited statistically significant differences compared to the GA groups (MD = −4.20 mg of oral morphine equivalent; 95% CI: −6.52 to −1.88, *P* = .0004, *I*^2^ = 97%). The forest plot data indicated that the SAPB + GA group did not significantly reduce opioid consumption^[20,21,27,28,30]^ at 48 hours (MD = −3.79 mg of oral morphine equivalent; 95% CI: −8.07 to 0.50, *P* = .08, *I*^2^ = 90%).

Subgroup analyses were conducted on 24 and 48-hour morphine intake based on study type due to the high heterogeneity. Among the 4 RCT studies, significant differences were observed between groups with low heterogeneity. However, no differences were found among the 4 cohort studies in 24 hours postoperative opioid consumption (RCTs: MD = −3.16, 95% CI: −5.55 to −0.76, *P* = .01, *I*^2^ = 38%; cohort studies: MD = −5.19, 95% CI: −11.48 to −1.10, *P* = .11, *I*^2^ = 98%). The difference in opioid consumption within 48 hours post-surgery between the 2 RCT studies and the 3 cohort studies was not statistically significant (RCTs: MD = −2.64, 95% CI: −14.78 to 9.49, *P* = .67, *I*^2^ = 63%; cohort studies: MD = −4.23, 95% CI: −9.17 to 0.72, *P* = .09, *I*^2^ = 94%) (Figure S3, Supplemental Digital Content, https://links.lww.com/MD/R516).

We stratified SAPB administration timing into preoperative and postoperative subgroups. Postoperative SAPB more effectively reduced 48-hour morphine consumption than preoperative delivery (Figure S4, Supplemental Digital Content, https://links.lww.com/MD/R516). In pediatric cohorts, 2 studies focused on postoperative opioids^[24,25]^: GA alone required significantly higher cumulative postoperative sufentanil and 24-hour post-extubation doses versus SAPB + GA.

### 3.6. Postoperative stress state

Furthermore, we assessed the postoperative stress response in both patient groups^[23,29,32]^ by measuring cortisol and interleukin levels. The forest plot indicated significant differences in cortisol levels between the 2 groups at the end of surgery and on postoperative day 1. However, no significant differences were observed in interleukin-6 (IL-6) and interleukin-10 (IL-10) levels at the end of surgery. Cortisol (at the end of surgery): MD = −41.68, 95% CI: −63.21 to −20.16, *P* = .0001, *I*^2^ = 64%; cortisol (postoperative day 1): MD = −35.43, 95% CI: −58.58 to −12.27, *P* = .003, *I*^2^ = 0%; IL-6: MD = −20.82, 95% CI: −103.76 to −62.11, *P* = .62, *I*^2^ = 91%; IL-10: MD = −1.31, 95% CI: −6.60 to −3.98, *P* = .63, *I*^2^ = 98%). This finding suggested that the levels of serum cortisol were lower in patients who received the SAPB compared to those in the GA group, which may contribute to enhanced postoperative recovery (Figure S5, Supplemental Digital Content, https://links.lww.com/MD/R516). Due to the limited sample size, subgroup analyses based on study type were not conducted.

### 3.7. Patients’ satisfaction

Only 1 study mentioned patient satisfaction with the analgesic effect, involving a total of 75 patients. Patient satisfaction with analgesia was assessed using a scoring system.^[21]^ The results indicated no significant difference in satisfaction between the SAPB + GA group (n = 38) and the GA group (n = 37) (*P* = .92).

### 3.8. Publication bias

In this meta-analysis, Egger test was employed to assess publication bias in the evaluation of the primary outcome indicators and total postoperative complications. The results of Egger test indicated a significant publication bias in the assessment of total postoperative complications, whereas no evidence of publication bias was found in the analyses of LOS and PONV (Table S2, Supplemental Digital Content, https://links.lww.com/MD/R516). Publication bias may occur due to the diminished accuracy resulting from a limited number of studies.

### 3.9. Sensitivity analysis

Sensitivity analysis, utilizing the leave-one-out method, was conducted for LOS, postoperative opioid consumption at 24 hours, the incidence of postoperative complications, and 24-hour VAS scores. The consistency of the estimates across these analyses affirmed the stability of the derived outcomes. However, our sensitivity analyses of patients’ postoperative opioid consumption at 48 hours indicated that the outcome lacked robustness (Figure S6, Supplemental Digital Content, https://links.lww.com/MD/R516).

## 4. Discussion

In this systematic review and meta-analysis, subgroup analyses stratified by age (adult vs pediatric patients) revealed that ultrasound-guided SAPB significantly reduced postoperative opioid consumption at both 24 and 48 hours. Among adults, SAPB correlated with reduced incidence of postoperative complications, including pruritus, hypoxemia, and pulmonary infection. Critically, no statistically significant intergroup difference in PONV incidence was observed. While SAPB did not reduce hospital LOS, it significantly decreased intensive care unit LOS following cardiac surgery compared to GA alone. Furthermore, SAPB + GA lowered postoperative pain scores at 12 and 24 hours and reduced serum cortisol levels, suggesting attenuated surgical stress and potentially enhanced recovery. These data support SAPB integration into multimodal analgesic regimens for cardiac surgery.

The 15 analyzed studies encompassed atrial/ventricular septal defect repairs, valve replacements, and coronary artery bypass grafting. Postoperative pain – ranging from moderate to severe – remains a clinical priority due to its potential to impede functional recovery. Although minimally invasive techniques have evolved for select procedures, sternotomy dependent surgeries still dominate, underscoring the imperative for optimized analgesic strategies.

Currently, opioids are predominantly administered intravenously. However, perioperative opioid use is often inadequate for effective pain management in cardiac surgery and is associated with a spectrum of potential side effects.^[37,38]^ Fascial-plane blocks have the potential to be advantageous elements within multimodal pain management strategies for cardiac surgery, with the objectives of improving patient comfort, facilitating earlier tracheal extubation, and promoting recovery and rehabilitation.^[39]^ The SAPB initially identified and introduced by Blanco, encompasses both deep and superficial approaches. The deep SAPB effectively anesthetizes the lateral cutaneous branches of the intercostal nerves at the T2 to T9 levels, while the superficial SAPB also provides effective blockade of the long thoracic (C5–7) and thoracodorsal (C6–8) nerves.^[40,41]^ Chu and Jarvis^[42]^ posited that SAPB has the potential to provide effective pain management in instances where postoperative analgesia from thoracic paravertebral block, epidural anesthesia, and intercostal nerve block proves inadequate. The judicious utilization of medical resources through the integration of traditional pain relief methods with fascial block aligns with the principles of enhanced recovery after surgery.^[43,44]^

The LOS in the hospital is a multifaceted outcome influenced by a variety of factors, including unpredictable fluctuations, such as changes in bed availability, hospital policies, and other unforeseen elements. The surgical incisions examined across all the fifteen studies, which encompassed both minimally invasive and open-heart surgeries, suggest that varying surgical techniques may influence the LOS. While no statistically significant difference emerged in overall hospital LOS, a clinically meaningful reduction in ICU LOS was observed. Subgroup stratification by study design revealed divergent outcomes: RCTs showed no ICU LOS difference, whereas cohort studies demonstrated significant reductions. These discrepancies may reflect methodological variance across study designs and institutional discharge protocols, necessitating validation through prospectively designed trials.

Our meta-analysis established that SAPB significantly reduced 24-hour postoperative opioid consumption and lowered pain scores versus GA alone, corroborating Aykut et al findings.^[27]^ Notably, this modest absolute reduction may lack clinical significance, underscoring the need for high-powered randomized trials. Significant heterogeneity (*I*^2^ > 50%) observed across studies likely reflects methodological divergence between observational and randomized designs.

However, no significant difference in opioid usage was observed at 48 hours post-surgery. This lack of variance may be attributed to the duration of the nerve block’s efficacy. As the drug anesthetic agent disperses over time, the effectiveness of the nerve block in providing pain relief diminishes. By optimizing the duration of regional analgesia tailored to specific surgical populations, there is an opportunity to reorient regional anesthesia research from primarily addressing immediate postoperative pain and 24 hours opioid consumption towards improving more clinically significant outcomes. Despite methodological variability in pain scales, SAPB + GA consistently reduced postoperative pain scores in both pediatric and adult cohorts (except for 48-hour NRS measurements). Evaluating pain intensity in pediatric populations is challenging due to their limited capacity to articulate and quantify discomfort relative to adults, further exacerbated by the lack of standardized pain assessment instruments, thereby complicating data collection.^[45]^

Moreover, our analysis revealed no increased adverse outcomes associated with SAPB. This finding aligns with systematic reviews by Hu and Zhang et al demonstrating SAPB’s safety profile across surgical contexts.^[46,47]^ Effective perioperative analgesia remains essential for reducing patient discomfort and potentially preventing postoperative complications. Notably, PONV incidence showed no significant difference between groups – consistent with prior research reporting no PONV reduction in thoracoscopic surgery patients.^[12]^ Furthermore, we observed no evidence suggesting elevated adverse event risk in SAPB recipients. No anesthesia-attributable perioperative mortality occurred.

A substantial stress response induces the secretion of various stress hormones, with cortisol serving as a specific and sensitive marker of stress intensity. IL-10 is recognized as an anti-inflammatory cytokine and a negative regulator of the immune response, playing a crucial role in maintaining cytokine equilibrium by inhibiting the production of IL-6.^[48]^ Our analysis compared serum cortisol levels between the 2 groups and found higher levels in the GA group. However, no significant differences in IL-6 or IL-10 levels were observed between the 2 groups. Due to the limited number of studies reporting inflammatory markers, the small sample size likely contributed to increased heterogeneity.

This meta-analysis is subject to certain limitations. Given the relatively recent application of SAPB in cardiac surgery, both RCTs and cohort studies were included to obtain sufficient evidence, potentially affecting the precision of the findings. Second, our analysis included diverse cardiac surgical procedures, which could influence postoperative pain outcomes; for example, open-heart surgery typically causes greater pain than minimally invasive approaches. Third, there was variation in the local anesthetic drugs and concentrations used across studies. Agents such as bupivacaine and ropivacaine were administered at varying concentrations. Standardizing local anesthetic regimens in future research would enhance the analysis of outcome measures.

The present study demonstrates that SAPB provides effective analgesia for cardiac surgical patients, significantly reducing postoperative opioid consumption and lowering the incidence of postoperative complications. Further research using robust methodological approaches is warranted to substantiate the efficacy and safety of SAPB for cardiac surgical procedures.

## Acknowledgments

The authors are grateful to the editor and all reviewers for their suggestions and help.

## Author contributions

**Conceptualization:** Xu-Bo Cui, Ming-Zhu Cui, Qiu-Ju Yang, Yun-Tai Yao.

**Data curation:** Xu-Bo Cui, Ming-Zhu Cui, Yang Liu, Yun-Tai Yao.

**Formal analysis:** Xu-Bo Cui, Ming-Zhu Cui, Yang Liu, Meng-Qi Yi, Xiao-Yang Zhang.

**Investigation:** Qiu-Ju Yang, Yun-Tai Yao.

**Methodology:** Xu-Bo Cui, Ming-Zhu Cui, Yang Liu, Meng-Qi Yi, Xiao-Yang Zhang.

**Project administration:** Qiu-Ju Yang, Yun-Tai Yao.

**Resources:** Qiu-Ju Yang, Yun-Tai Yao.

**Supervision:** Qiu-Ju Yang, Yun-Tai Yao.

**Software:** Xu-Bo Cui, Ming-Zhu Cui, Yang Liu, Meng-Qi Yi, Xiao-Yang Zhang.

**Validation:** Xu-Bo Cui, Ming-Zhu Cui, Yang Liu.

**Visualization:** Xu-Bo Cui, Ming-Zhu Cui, Yang Liu.

**Writing – original draft:** Xu-Bo Cui, Ming-Zhu Cui.

**Writing – review & editing:** Xu-Bo Cui, Ming-Zhu Cui, Yun-Tai Yao.

## Supplementary Material
