# Supplementary material for: The efficacy and safety of serratus anterior plane block in patients undergoing cardiac surgery: A systematic review and meta-analysis
Source: Medicine (Baltimore). 2026 Mar 13;105(11):e48013. doi: 10.1097/MD.0000000000048013 (PMC12991781; doi:10.1097/MD.0000000000048013)
Supplement: Supplementary file 1 [file medi-105-e48013-s001.docx]

| Search strategies | Results |
| --- | --- |
| PubMed  ((((((((((((((((Cardiac Surgical Procedures) OR (Surgical Procedure, Cardiovascular)) OR (Procedure, Cardiovascular Surgical)) OR (Cardiovascular Surgical Procedure)) OR (Procedures, Cardiovascular Surgical)) OR (Surgical Procedures, Cardiovascular)) OR ((((((Coronary Artery Bypass) OR (Artery Bypass, Coronary)) OR (Artery Bypasses, Coronary)) OR (Bypasses, Coronary Artery)) OR (Coronary Artery Bypasses)) OR (Coronary Artery Bypass Surgery))) OR (Extracorporeal Circulation)) OR (Cardiopulmonary Bypass)) OR (((((Heart Diseases) OR (Cardiac Diseases)) OR (Cardiac Disease)) OR (Cardiac Disorders)) OR (Cardiac Disorder))) OR (Cardiopulmonary Bypass)) OR (Heart Valve Diseases)) OR (Aortic Valve Disease)) OR (Myocardial Ischemia)) OR (Rheumatic Heart Disease)) OR (Coronary Disease)) AND (serratus anterior plane block) | 44 |
| Embase  'Serratus anterior plane block' AND (('cardiac'/exp OR cardiac) AND surgical AND ('procedures'/exp OR procedures) OR 'surgical procedure, cardiovascular': ab, ti OR 'procedure, cardiovascular surgical': ab, ti OR 'cardiovascular surgical procedure': ab, ti OR (('procedures, cardiovascular surgical': ab, ti OR coronary OR artery) AND bypass) OR 'artery bypass, coronary': ab, ti OR 'artery bypasses, coronary': ab, ti OR 'bypasses, coronary artery': ab, ti) | 25 |
| Cochrane  (Cardiovascular Surgical Procedures) OR (Cardiopulmonary Bypass) OR (Extracorporeal Circulation) OR (Coronary Artery Bypass) OR (Heart Diseases) OR (Heart Valve Diseases) OR (Surgical Procedure, Cardiovascular) OR (Procedure, Cardiovascular Surgical) OR (Cardiovascular Surgical Procedure) OR (Procedures, Cardiovascular Surgical) OR (Surgical Procedures, Cardiovascular) OR (Heart-Lung Bypass) OR (Bypass, Heart-Lung) OR (Bypasses, Heart-Lung) OR (Heart Lung Bypass) OR (Heart-Lung Bypasses) OR (Bypass, Cardiopulmonary) OR (Bypasses, Cardiopulmonary) OR (Cardiopulmonary Bypasses) OR (Circulation, Extracorporeal) OR (Circulations, Extracorporeal) OR (Extracorporeal Circulations) OR (Artery Bypass, Coronary) OR (Artery Bypasses, Coronary) OR (Bypasses, Coronary Artery) OR (Coronary Artery Bypasses) OR (Heart Disease) OR (Cardiac Diseases) OR (Cardiac Disease) OR (Cardiac Disorders) OR (Cardiac Disorder) OR (Heart Disorders) OR (Heart Disorder) OR (Heart Valve Disease) OR (Valve Disease, Heart) OR (Heart Valvular Disease) OR (Disease, Heart Valvular) OR (Heart Valvular Diseases) OR (Valvular Disease, Heart) OR (Valvular Heart Diseases) OR (Heart Disease, Valvular) OR (Valvular Heart Disease) in Title Abstract Keyword AND (serratus anterior plane block) OR (serratus anterior block) in Title Abstract Keyword | 58 |
| Web of Science  (TS=((Cardiovascular Surgical Procedures) OR (Cardiopulmonary Bypass) OR (Extracorporeal Circulation) OR (Coronary Artery Bypass) OR (Heart Diseases) OR (Heart Valve Diseases) OR (Surgical Procedure, Cardiovascular) OR (Procedure, Cardiovascular Surgical) OR (Cardiovascular Surgical Procedure) OR (Procedures, Cardiovascular Surgical) OR (Surgical Procedures, Cardiovascular) OR (Heart-Lung Bypass) OR (Bypass, Heart-Lung) OR (Bypasses, Heart-Lung) OR (Heart Lung Bypass) OR (Heart-Lung Bypasses) OR (Bypass, Cardiopulmonary) OR (Bypasses, Cardiopulmonary) OR (Cardiopulmonary Bypasses) OR (Circulation, Extracorporeal) OR (Circulations, Extracorporeal) OR (Extracorporeal Circulations) OR (Artery Bypass, Coronary) OR (Artery Bypasses, Coronary) OR (Bypasses, Coronary Artery) OR (Coronary Artery Bypasses) OR (Heart Disease) OR (Cardiac Diseases) OR (Cardiac Disease) OR (Cardiac Disorders) OR (Cardiac Disorder) OR (Heart Disorders) OR (Heart Disorder) OR (Heart Valve Disease) OR (Valve Disease, Heart) OR (Heart Valvular Disease) OR (Disease, Heart Valvular) OR (Heart Valvular Diseases) OR (Valvular Disease, Heart) OR (Valvular Heart Diseases) OR (Heart Disease, Valvular) OR (Valvular Heart Disease))) AND TS=((serratus anterior plane block) OR (serratus anterior block)) | 17 |
| CKNI、Wan Fang  ((serratus anterior plane block) [All field] OR (serratus anterior block) [All field]) AND ((cardiovascular Surgical Procedures) [All field] OR (coronary artery bypass) [All field] OR (valvular heart disease) [All field] OR (extracorporeal Circulation) [All field]) | 19 |
| Other Sources | 4 |
