# Supplementary material for: The efficacy and safety of serratus anterior plane block in patients undergoing cardiac surgery: A systematic review and meta-analysis
Source: Medicine (Baltimore). 2026 Mar 13;105(11):e48013. doi: 10.1097/MD.0000000000048013 (PMC12991781; doi:10.1097/MD.0000000000048013)

**Supplementary Table 1.** Opioids dosage conversion form

| Morphine  iv | Tramadol  iv | Oxycodone  po | Morphine  po | Fentanyl  iv | Sufentanil  iv |
| --- | --- | --- | --- | --- | --- |
| iv = 10mg | iv = 100mg | po = 20mg | po = 30mg | iv = 100μg | iv = 10μg |

**Supplementary Table 2.** Egger’s test

| Outcome | t | *P* |
| --- | --- | --- |
| LOS in the hospital | -0.74 | 0.489 |
| LOS in the ICU | -0.35 | 0.742 |
| Postoperative complications | -2.38 | 0.045 |
| PONV | -2.07 | 0.072 |

LOS: length of stay, ICU: intensive care unit, PONV: postoperative nausea and vomiting.

Supplementary Figures:

**Supplementary Fig. 1** Forest plot of patients’ baseline characteristics


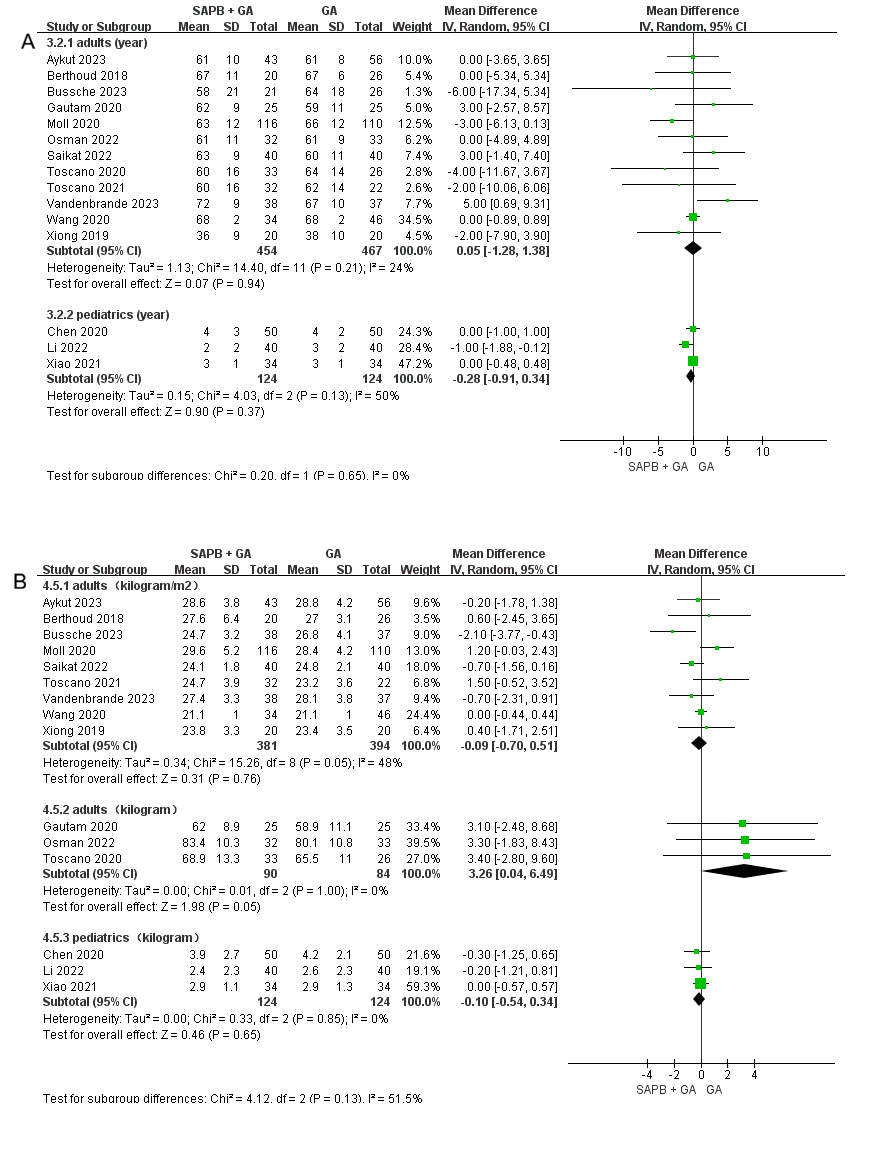


**Supplementary Fig. 2** Subgroup analysis by study type for length of stay in the intensive care unit


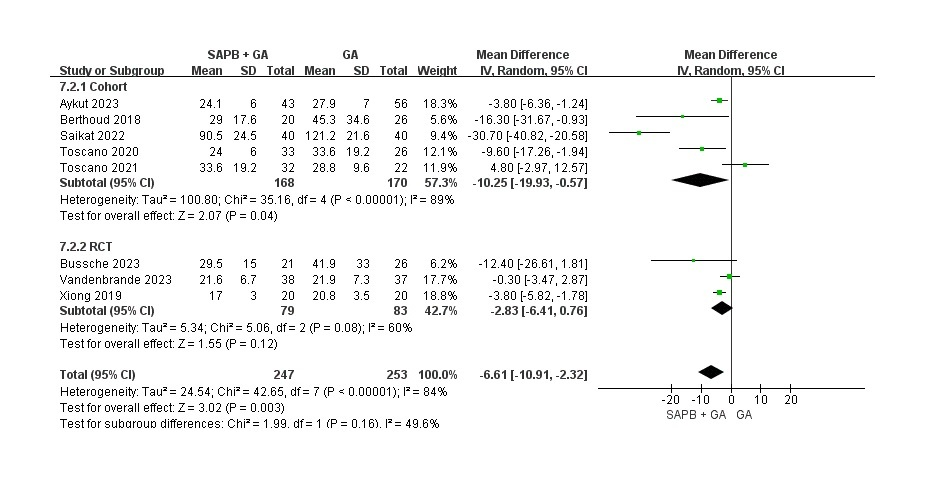


**Supplementary Fig. 3** Subgroup analysis by study type for postoperative opioid consumption: 24h postoperative opioid consumption (**A**)**;** 48h postoperative opioid consumption (**B**)

**
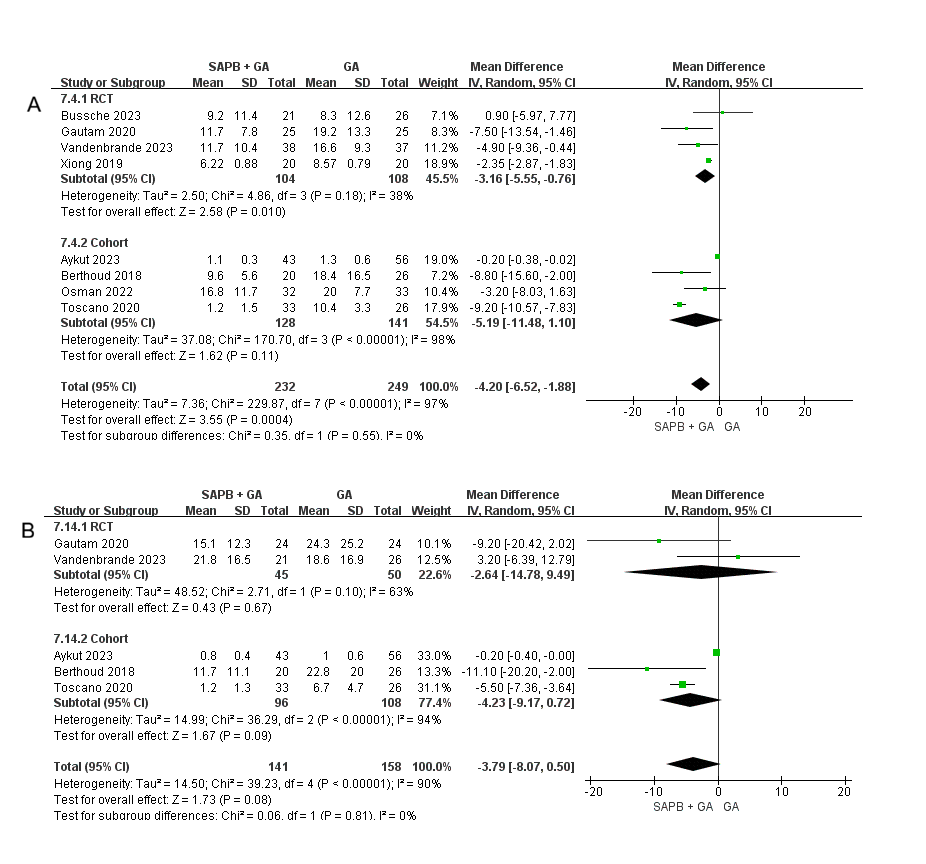
**

**Supplementary Fig. 4** Subgroup analysis stratified by SAPB administration timing: 24h postoperative opioid consumption (**A**)**;** 48h postoperative opioid consumption (**B**)


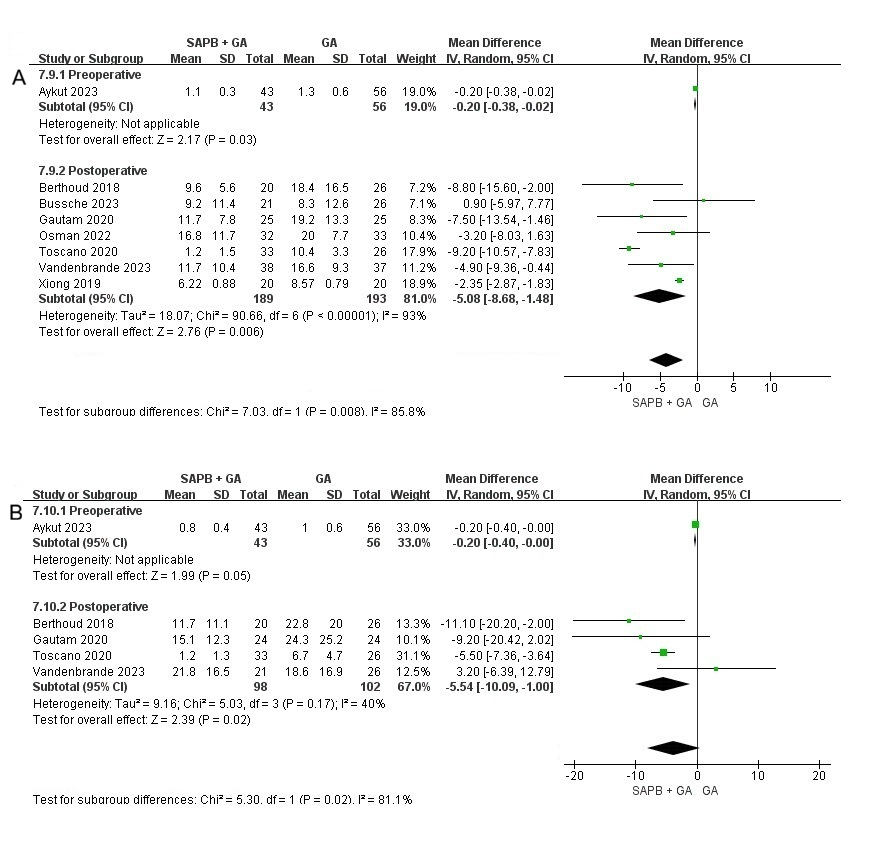


**Supplementary Fig. 5** Forest plot of cortisol, interleukin-6 and interleukin-10


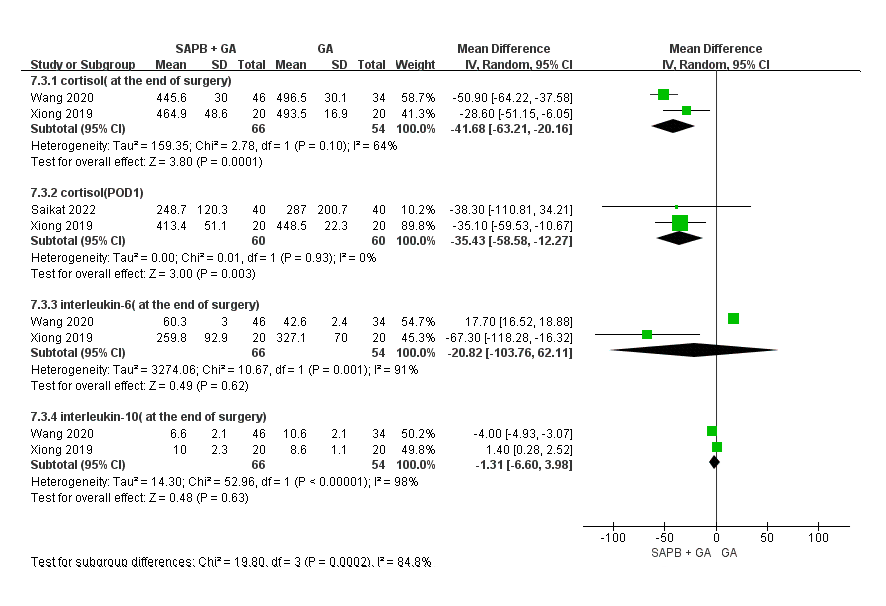


**Supplementary Fig. 6** Sensitivity analysis. Length of stay in the hospital (**A**); Length of stay in intensive care unit (**B**); Adults’ 24h static pain visual analogue scale scores (**C**); The incidence of postoperative complications (**D**); The incidence of postoperative nausea and vomiting (**E**); The 24h postoperative opioid consumption (**F**); The 48h postoperative opioid consumption (**G**)


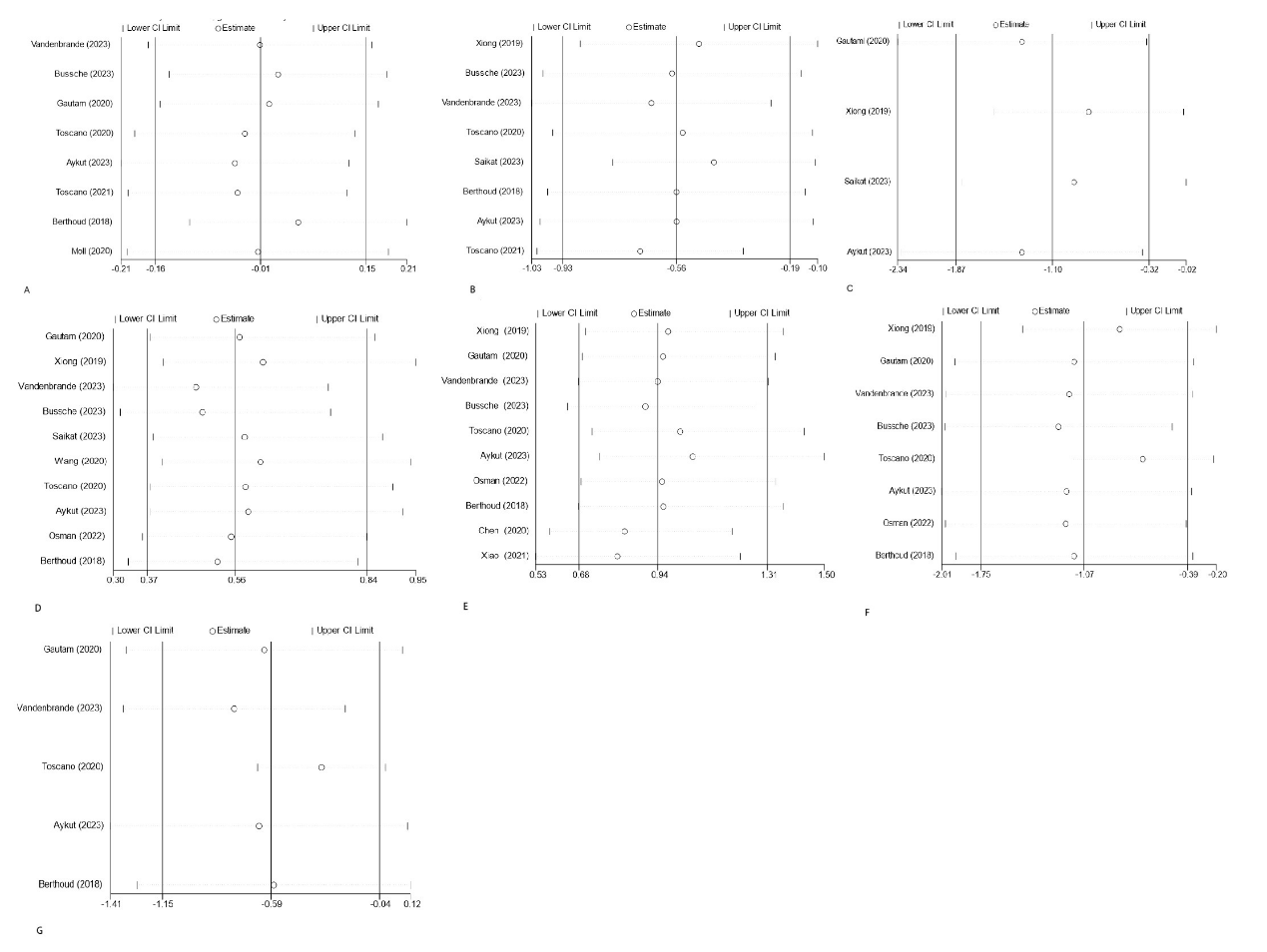

Supplement: Supplementary file 2 [file medi-105-e48013-s002.docx]
